# *Mycobacterium orygis*–Associated Tuberculosis in Free-Ranging Rhinoceros, Nepal, 2015

**DOI:** 10.3201/eid2203.151929

**Published:** 2016-03

**Authors:** Jeewan Thapa, Sarad Paudel, Amir Sadaula, Yogendra Shah, Bhagwan Maharjan, Gretchen E. Kaufman, Deborah McCauley, Kamal P. Gairhe, Toshio Tsubota, Yasuhiko Suzuki, Chie Nakajima

**Affiliations:** Hokkaido University, Sapporo, Japan (J. Thapa, S. Paudel, Y. Shah, T. Tsubota, Y. Suzuki, C. Nakajima);; National Trust for Nature Conservation, Biodiversity Conservation Center, Chitwan, Nepal (A. Sadaula);; German Nepal Tuberculosis Project, Kathmandu, Nepal (B. Maharjan);; Veterinary Initiative for Endangered Wildlife, Bozeman, Montana, USA (G.E. Kaufman, D. McCauley);; Chitwan National Park Department of National Parks and Wildlife Conservation, Chitwan (K.P. Gairhe)

**Keywords:** greater one-horned rhinoceros, Rhinoceros unicornis, Indian rhinoceros, free-ranging rhinoceros, Mycobacterium orygis, Mycobacterium tuberculosis complex, tuberculosis, tuberculosis and other mycobacteria, bacteria, Nepal

**To the Editor:** *Mycobacterium orygis*, previously described as oryx bacilli, has recently been categorized as a member of *M. tuberculosis* complex and has been reported to cause tuberculosis (TB) in a variety of animals and in humans. Most reported isolates were of South Asian origin (*1*). In a previous study (*2*), we isolated and molecularly characterized *M. orygis* isolates from wild animals living in a captive facility in Kathmandu, Nepal.

The greater one-horned rhinoceros (*Rhinoceros unicornis*), or Indian rhinoceros, is the largest species of rhinoceros. It is listed in Appendix I of the Convention on International Trade in Endangered Species (https://cites.org/eng/app/appendices.php), designated as vulnerable by the International Union for Conservation of Nature Red List (http://www.iucnredlist.org/search), and designated as a protected species by the Government of Nepal (*3*). Because of successful conservation efforts, the current wild population of greater one-horned rhinoceros in Nepal and India has increased from 600 in 1975 to 3,555 in mid-2015 (*4*). As of 2015, the population of these rhinoceros in Nepal was 645, including 605 animals living in Chitwan National Park (CNP) (*5*).

On February 16, 2015, CNP officials observed a sick female rhinoceros in the buffer zone of the western sector of the park near Amaltari. The rhinoceros was dull, depressed, and not feeding. The following day, the animal was found dead in the same area (Technical Appendix Figure 1). Superficial maggot-infested wounds were on both sides of the vulva, indicating that the rhinoceros was not able to naturally remove the maggots and suggesting that the animal was sick for some time. During the necropsy, several granulomatous lesions were observed in the lungs and considered to be compatible with TB infection. The lesions were extensively distributed and well encapsulated and contained caseous necrotic material (Technical Appendix Figure 2). No other pathologic changes were observed in any of the organs examined, leading to the conclusion that the rhinoceros died from TB.

A lung tissue sample positive for TB by acid-fast staining was cultured on Lowenstein-Jensen media. We performed spoligotyping and mycobacterial interspersed repetitive units–variable-number tandem-repeat (MIRU-VNTR) procedure on the isolate as previously described (*6*,*7*). Spoligotyping analysis, performed as previously described (*2*), showed that the isolate had a spoligo–international type 587 pattern, indicating it was *M. orygis*. We also performed multilocus sequence typing on various genes (*2*), and confirmed that the isolate was *M. orygis*. We then constructed a dendrogram by comparing the MIRU-VNTR result from rhinoceros isolate with published *M. orygis* MIRU-VNTR types (Figure) (*1*,*2*,*8*). The rhinoceros *M. orygis* isolate fell in a unique position in the dendrogram; we identified a difference in only 1 locus (MIRU 424) when we compared the isolate with the largest cluster of reported *M. orygis* isolates, including those previously reported from Nepal.

**Figure:**
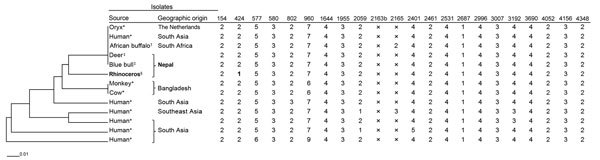
Phylogeny of *Mycobacterium orygis* isolates as determine on the basis of mycobacterial interspersed repetitive units–variable-number tandem-repeat (MIRU-VNTR) results of 22 loci. The unweighted pair group method with arithmetic mean dendrogram was drawn by using MIRU-VNTR*plus* software (http://www.miru-vntrplus.org). The order of MIRU-VNTR is as follows, left to right: 154, 424, 577, 580, 802, 960, 1644, 1955, 2059, 2163b, 2165, 2401, 2461, 2531, 2687, 2996, 3007, 3192, 3690, 4052, 4156 and 4348. *Isolates from (*1*), †isolate from (*8*), ‡isolates from (*2*), §isolate from this study. Bold MIRU-VNTR copy number of locus 424 in rhinoceros isolate indicates a single locus difference in MIRU-VNTR type from the largest cluster. X, unamplifiable. Scale bar indicates genetic distance

In our earlier study (*2*), we isolated *M. orygis* from chital deer (*Axis axis*) and blue bull (*Boselaphus tragocamelus*) from a captive wild-animal facility and postulated that the origin of the infection might be from infected animals in CNP, where the deer and blue bull originated. This new finding of a different strain type of *M. orygis* in a free-ranging rhinoceros in CNP provides evidence for our hypothesis. Other reports of *M. orygis* in captive wild animals in Nepal (*2*), cattle and a rhesus monkey in Bangladesh (*1*), humans in South Asia (*1*), and an immigrant from India in New Zealand (*9*) further support this bacterium’s potential widespread distribution in South Asia and attests to the One Health significance of this organism.

In a demographic study of rhinoceros in Nepal (*10*), the animals were found to be living in a narrow area of riverine grassland in CNP. A chronic and devastating disease like TB in this vulnerable and isolated population, which is already threatened from habitat destruction and poaching, is a matter of great conservation concern for the animal’s long-term survivability. Also, CNP is listed by the United Nations Educational, Scientific and Cultural Organization as a World Heritage site because of its rich biodiversity and as an important habitat for endangered animals, including Bengal tigers (*Panthera tigris*) and Asian elephants (*Elephas maximus*). Thus, *M. orygis*–associated TB in rhinoceros in CNP may also indicate a threat to other animals, including some that are endangered. There is a strong possibility of unknown maintenance hosts of *M. orygis* in and around the national park. Our findings support the need for further investigation to understand the ecology and epidemiology of *M. orygis* and provide justification for active surveillance of this bacterium in animals in the national park and in livestock and humans in the buffer-zone areas. Furthermore, the increasing evidence for widespread distribution of *M. orygis* in South Asia provides a new picture of TB and may lead to a new understanding of *M. tuberculosis* complex.

Technical Appendix Location in Nepal where *Mycobacterium orygis*–infected rhinoceros was found dead and image of granulomatous tuberculosis lesion.
