# Supplementary material for: Mycobacterium orygis–Associated Tuberculosis in Free-Ranging Rhinoceros, Nepal, 2015
Source: Emerg Infect Dis. 2016 Mar;22(3):570–2. doi: 10.3201/eid2203.151929 (PMC4766909; doi:10.3201/eid2203.151929)
Supplement: Technical Appendix — Location in Nepal where Mycobacterium orygis–infected rhinoceros was found dead and image of granulomatous tuberculosis lesion. [file 15-1929-Techapp-s1.pdf]

Article DOI: http://dx.doi.org/10.3201/eid2203.151929

Tuberculosis Caused by *Mycobacterium orygis* in Free-Ranging Rhinoceros, Nepal, 2015

Technical Appendix

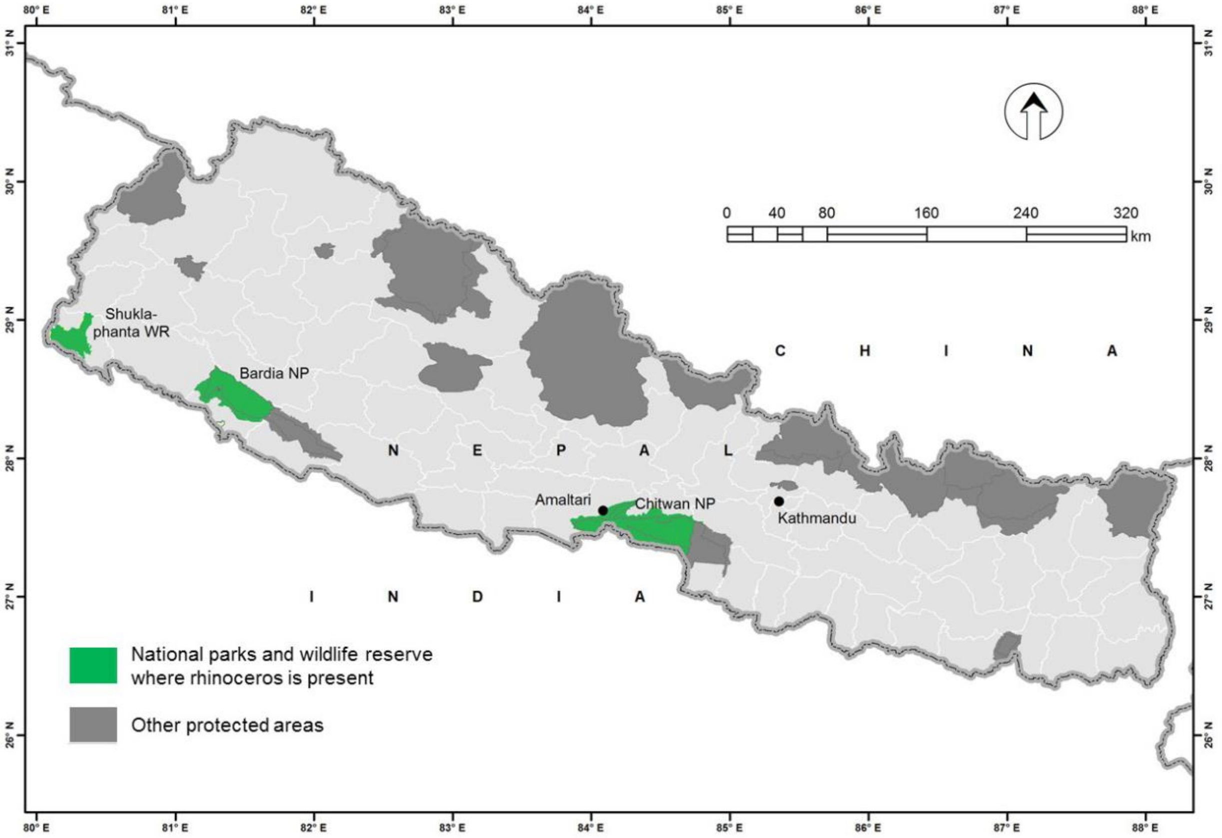

Technical Appendix Figure 1. Location of Amaltari in Chitwan National Park from where rhinoceros was found dead. Kathmandu is place of captive facility from where *Mycobacterium orygis* was reported from wild animals.

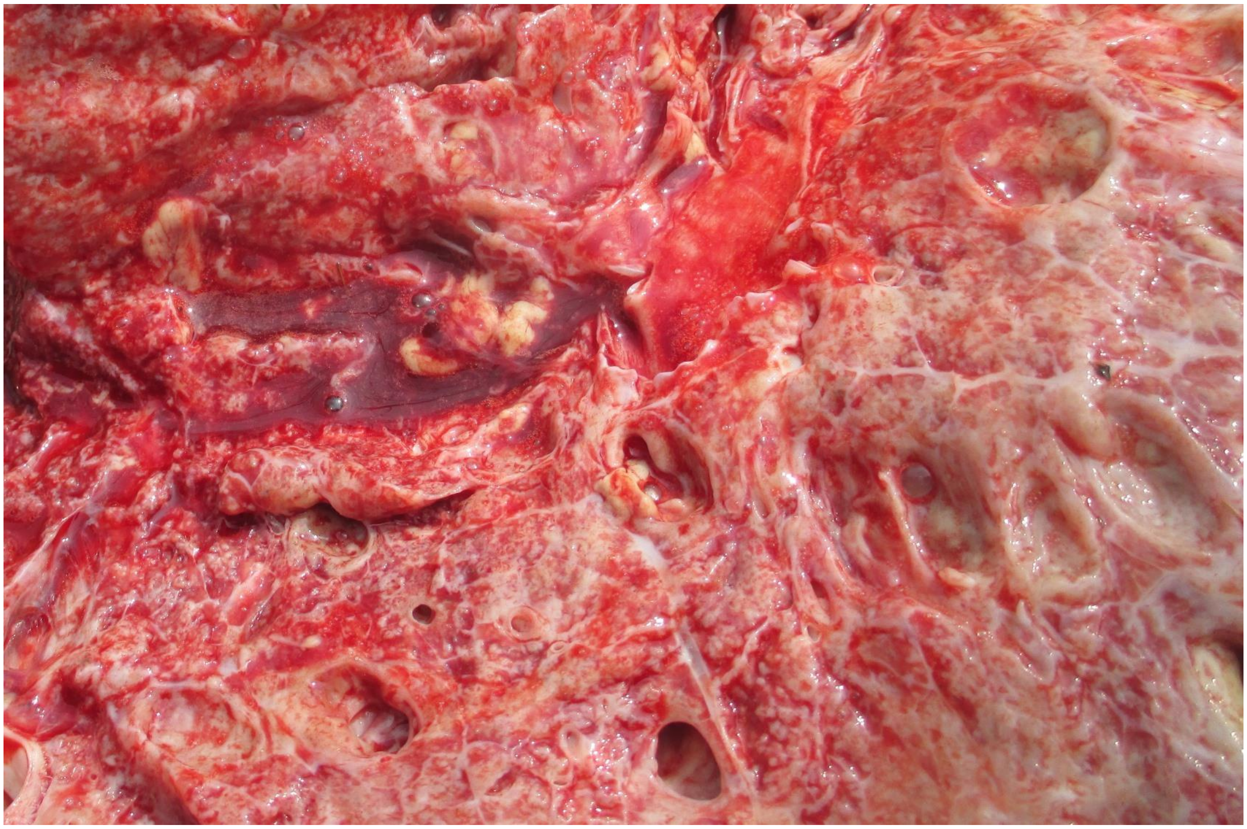

Technical Appendix Figure 2. Granulomatous tuberculosis lesion with caseous mass in lungs.
